# The Medical, Surgical, and Hygienic Exhibition, 1901

**Published:** 1901-06-29

**Authors:** 


### THE MEDICAL, SURGICAL, AND HYGIENIC EXHIBITION, 1901.

(*Concluded from page* 186.)

The Protene Company, Limited, 36 Welbeck Street, W., exhibited their milk-proteid manufactures. Protene is prepared in many convenient forms, such as bread, biscuits, chocolate, and diabetic foods. Special prominence was given to the diabetic foods, and to their new productions the Saclac, the Regent, and the Welbeck biscuits, which contain 25 per cent. of milk proteids. International Plasmon, Limited, 56 Duke Street, Grosvenor Square, W., gave an interesting show of their plasmon manufactures, including powder, cocoa, biscuits, chocolate, and beef extract. Plasmon cocoa deserves special mention as claiming to possess high nourishing qualities. The British Somatose Company, Limited, 165 Queen Victoria Street, E.C., exhibited their somatose (made from beef), iron somatose, and milk somatose. A new preparation of somatose, in liquid form, was shown, which is easy of administration. Somatose is a nourishing food for invalids and convalescents.

Messrs. G. Van Abbott and Sons, 104 Wigmore Street, W., gave a varied exhibit of their foods for diabetics, dispeptics, and invalids, including gluten bread, soya bread, biscuits, cakes, jellies, and soups. This firm has reached a high degree of excellence in their manufactures. Messrs. Callard and Co., 65 Regent Street, W., had an extensive exhibit of food for the diabetic, for reducing weight, for the rheumatic and gouty, and for the dyspeptic. Specimens were shown of spinach biscuits and celery biscuits, in which the flavour of the vegetables is preserved. The Manhu Food Company, Limited, Liverpool, showed their Manhu diabetic foods. The feature of these food preparations is the change which is said to take place in the starch, and by which digestion and assimilation are facilitated. "Cheltine" Foods, Limited, Cheltenham, had a prominent show of "Cheltine" foods for the diabetic, the anæmic, the dyspeptic, and for invalids and infants. It is claimed for the diabetic preparations that the starch is so treated as to serve its normal purpose in the nutrition of the body. The Hovis-Bread Flour Company, Limited, Macclesfield, exhibited their bread, flour, and biscuits, and also "Lito," a new self-raising flour.

Messrs. Brand and Co., Limited, Mayfair, W., displayed their high-class invalid specialities, including meat essences, peptones, and jellies and soups put up in bottles, instead of tins as formerly. Particular attention was drawn to their fever food which is recommended for enteric and other fevers, and to their nutrient powder which is a valuable article of diet in wasting diseases. At the stall of Messrs. Cosenza and Co., 95 Wigmore Street, W., examples of Maggi's consommé tonic bouillon, and cross star soups were exhibited. These preparations have been largely supplied for hospital use in South Africa. An apparatus was shown for the treatment of phthisis by the vapours of Igazol, together with specimens of Igazol. Tansan, a Japanese natural tonic table water, was also shown at this stand. Bovril, Limited, 152 Old Street, E.C., displayed a variety of their bovril preparations, including extracts of beef and liquor carnis (raw meat juice). The adjoining stall was that of Virol, Limited, 152 Old Street, E.C., on which was shown "Virol," which consists of the proteids fats and salts of beef and eggs, beef marrow and malt extract. At this stall a list was exhibited of hospitals where this preparation has been used. Messrs. Liebig's Extract of Meat Company, Limited, 9 Fenchurch Avenue, E.C., gave an attractive display of "Lemco," the renowned extract of beef, Peptarnis—peptone of beef—and their new meat beverage, "Oxo," which contains a high percentage of the essential properties of beef. The cost of "Oxo" being low its introduction to hospitals would effect considerable economy. Messrs. G. Nelson, Dale and Co., Limited, 14 Dowgate Hill, E.C., gave an exhibit of "Hipi," a mutton essence, which is an agreeable change from beef extracts in the diet of invalids. It has been found an excellent article of diet in cases of gastric ulcer. Messrs. J. G. Cox, Limited, Eastcheap Buildings, E.C., exhibited their sparkling gelatine, which is asserted to be free from chemicals, and therefore safe for administering with wine, beef tea, &c. Cerebos, Limited, Newcastle-on-Tyne, had a large display of Cerebos salt, which contains the mixed phosphates as found in wheaten bran, and is therefore a valuable dietetic.

Messrs. Cadbury Brothers, Limited, Bournville, near Birmingham, exhibited their cocoa, which is renowned for its purity.

Messrs. Marshall and Elvy, Limited, 45 New Oxford Street, W.C., gave an exhibit of Courvoisier's brandy, which enjoys a high reputation, and is specially suited for medical purposes; and Satinette, a new stimulant resembling gin, which is doubly distilled and matured by age. Messrs. Stephen Smith & Co., Bow, E., displayed Hall's wine, Keystone burgundy, and Keystone beef wine, which are already familiar to the public. South Australian wines, of the "Orion" brand, were shown. These wines have attained a considerable sale in this country, and their purity is guaranteed by the certificate of the Government of South Australia.

A variety of temperature charts were shown by Messrs. Wodderspoon and Co., 7 Searle Street, W.C., who are well known among the members of the medical and nursing professions in this connection.

